# A Hypothesis Regarding the Molecular Mechanism Underlying Dietary Soy-Induced Effects on Seizure Propensity

**DOI:** 10.3389/fneur.2014.00169

**Published:** 2014-09-03

**Authors:** Cara Jean Westmark

**Affiliations:** ^1^Department of Neurology, Medical Sciences Center, University of Wisconsin, Madison, WI, USA

**Keywords:** autism, daidzein, estrogen, fragile X syndrome, mGluR_5_, phytoestrogens, seizures, soy

## Abstract

Numerous neurological disorders including fragile X syndrome, Down syndrome, autism, and Alzheimer’s disease are co-morbid with epilepsy. We have observed elevated seizure propensity in mouse models of these disorders dependent on diet. Specifically, soy-based diets exacerbate audiogenic-induced seizures in juvenile mice. We have also found potential associations between the consumption of soy-based infant formula and seizure incidence, epilepsy comorbidity, and autism diagnostic scores in autistic children by retrospective analyses of medical record data. In total, these data suggest that consumption of high levels of soy protein during postnatal development may affect neuronal excitability. Herein, we present our theory regarding the molecular mechanism underlying soy-induced effects on seizure propensity. We hypothesize that soy phytoestrogens interfere with metabotropic glutamate receptor signaling through an estrogen receptor-dependent mechanism, which results in elevated production of key synaptic proteins and decreased seizure threshold.

## Introduction

Soy was first grown in the United States in the 1760s. This leguminous plant is native to East Asia and related to peas, alfalfa, and clover. Soybeans are the seeds of the soy plant and contain high levels of protein with significant quantities of the essential amino acids. Soybeans are the only plant source of complete protein containing all 20 amino acids. Soybeans are ground to produce soybean oil and soybean meal. Soybean oil is used in food products such cooking oil and in industrial products including plastics and biodiesel fuel. Soybean meal, the protein-rich fraction, is used in food, beverages, and condiments for human consumption as well as in animal feed. According to the American Soybean Association, soybeans were planted on over 75 million acres in the United States in 2012 and were the second largest cash crop with over 3 billion bushels produced. This vegetable protein is used extensively by the food industry in the United States as an additive in nearly all types of foods. Soy protein is also sold as a dietary supplement and is the key ingredient in soy-based infant formulas.

Soy has been purported as a dietary therapy for cardiovascular disease, type 2 diabetes, osteoporosis, hormone-dependent cancers, and the symptoms of menopause, but medical research has been inconclusive in substantiating many of these claims. The FDA recommends that 25 g/day of soy protein, as part of a diet low in saturated fat and cholesterol, may reduce the risk of heart disease; however, the Nutrition Committee of the American Heart Association reviewed 22 randomized clinical trials comparing isolated soy protein containing isoflavones to milk and other proteins on LDL-cholesterol levels and found the average effect was only 3% (1). They found no benefit regarding HDL cholesterol, triglycerides, or blood pressure. A subsequent meta-analysis found a 4.2–5.5% reduction in LDL cholesterol, a 3.2% increase in HDL cholesterol, and a 10.7% decrease in fasting triacylglycerol levels suggesting that daily consumption of 15–30 g of soy significantly improved serum risk factors for cardiovascular disease (2). A meta-analysis of soy product consumption in patients with type 2 diabetes mellitus found that soy protein intake was beneficial in diabetic patients in terms of serum lipids, but there were no significant effects on fasting glucose, insulin, or glycated hemoglobin levels (3). Osteoporosis studies indicate that soy isoflavones stimulate bone formation, inhibit bone resorption, and increase bone mineral density, resulting in attenuation of bone loss in menopausal women (4–9), albeit there are reports of only slight or no clinical effects (10). Cancer meta-analyses indicate that the consumption of soy or soy isoflavones is associated with reduced prostate cancer (11–14), gynecological cancers (15), and possibly breast cancer (16–21). There is no conclusive evidence that soy phytoestrogens reduce hot flashes associated with menopause (22–24). Overall, the literature contains many conflicting reports regarding the health benefits of consuming soy and phytoestrogen supplements. While the FDA has authorized a health claim linking the consumption of soy protein with a reduced risk of coronary heart disease, the agency also lists soy in its poisonous plant database with warnings regarding goiter, growth problems, amino acid deficiencies, mineral malabsorption, endocrine disruption, and carcinogenesis (25).

Perhaps the most controversial use of soy is in soy-based infant formulas. The current position of the American Academy of Pediatrics is, “There is no conclusive evidence from animal, adult human, or infant populations that dietary soy isoflavones may adversely affect human development, reproduction, or endocrine function (26).” And the national toxicology program (NTP) Center for the Evaluation of Risks to Human Reproduction (CERHR) found that, “The overall evidence was considered insufficient to reach a conclusion on whether the use of soy infant formula produces or does not produce developmental toxicity with infant exposure in girls or boys at recommended intake levels” (27). In other words, there is not conclusive evidence that soy-based infant formulas are safe. Based on market sales, 12% of infant formulas in the United States are soy-based (27). Approximately 20–25% of infants receive some soy-based formula during their first year, but there is no data regarding how many are exclusively fed soy-based formula (28). While there may be health benefits for adults associated with the consumption of soy, this natural product holds potential danger for children. Soy products are rich in phytoestrogens, which are natural plant chemicals that have estrogenic and anti-estrogenic properties. The effects of phytoestrogens on fetal and early childhood development have not been extensively studied (29–31). Rodent studies indicate that the placenta acts as a sink for phytoestrogens, and that while transport of phytoestrogens across the placenta is inefficient, low levels are found in the fetus and are sufficient for activation of estrogen receptor beta (ERβ) (32). A 4-month-old infant fed soy formula would be exposed to 4.5–8 mg/kg/day of soy phytoestrogens (33, 34), which is 6–11 times the dose necessary to exert hormone-like effects in adults (33). In placental mammals, the fetus is continuously exposed to high levels of estrogen from the placenta and the mother. Environmental exposure to phytoestrogens during this period is expected to disrupt the function of the natural steroid hormones.

In summary, the safety of long-term soy phytoestrogen consumption remains a controversy. Our research, which is reviewed below, suggests that soy-based diets are associated with increased seizure susceptibility in both rodent and human models of neurological disease. In this Hypothesis and Theory paper for the Diet and Brain Disorders Research Topic of Frontiers in Neurology, we consider the potential molecular mechanism underlying soy-associated effects on seizure propensity. We hypothesize that soy phytoestrogens interfere with metabotropic glutamate receptor (mGluR) signaling through an estrogen receptor (ER)-dependent mechanism, which results in elevated production of key synaptic proteins and decreased seizure threshold in genetically susceptible individuals. First, we summarize our recent research, which implicates soy protein consumption with increased seizure susceptibility. Second, we discuss the potential implications of these findings for infants fed soy-based infant formulas. Third, we review published work in the areas of fragile X syndrome (FXS), mGluR_5_ signaling, and ER signaling that forms the foundation for our hypothesis. Fourth, we present our working model regarding the molecular mechanism underlying soy-induced seizure activity. And finally, we discuss alternative hypotheses that could explain soy-induced health effects.

## Soy Consumption, Seizures, and Autism

**Figure 1:**
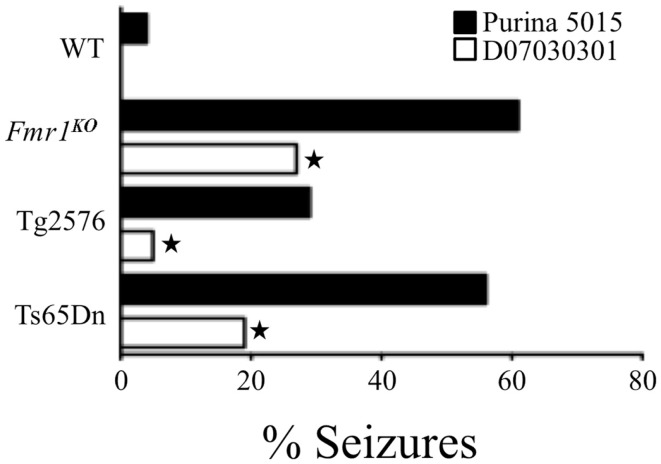
**Soy-free diet reduces seizure propensity in several strains of mice including *Fmr1^KO^* (FXS mouse model that lacks FMRP expression), Tg2576 (Alzheimer’s disease mouse model that over-expresses the human *APP_695_* gene with the Swedish familial mutation), and Ts65Dn (Down syndrome mouse model that is trisomic for chromosome 16 carrying the *App* gene) (38)**. These mouse models of neurological disease and WT littermates were conceived and maintained on soy-based Purina 5015 chow. At age P18, pups were left on the Purina 5015 (black bars) or transferred to a purified ingredient, soy-free diet (D07030301, white bars) for 3 days prior to seizure testing. Statistical significance between mice of the same genotype but fed different diets was determined by Barnard’s exact test (two-tail) and is denoted by a star (*P* ≤ 0.05).

Our research has examined audiogenic-induced seizure (AGS) incidence after chronic treatment with mGluR_5_ antagonists in several transgenic mouse lines (35, 36). In pursuit of these objectives, we incorporated the mGluR_5_ antagonist fenobam into a purified ingredient, soy-free diet that was matched to our standard lab chow (Purina 5015) for protein, fat, and carbohydrate content. We chose to incorporate the drug into a soy-free diet because Purina lab chows are grain based and nutrients vary from lot to lot (37). Surprisingly, chronic feeding with the soy-free diet alone (no fenobam added) (D07030301) for 3 days prior to seizure testing drastically attenuated AGS in multiple mouse lines including *Fmr1^KO^* (FXS), Tg2576 (Alzheimer’s disease), and Ts65Dn (Down syndrome) (38) (Figure 1). We hypothesized that a longer treatment period might further reduce seizure susceptibility and assessed seizures in *Fmr1^KO^* mice conceived and maintained on D07030301 until AGS testing at postnatal day 21. We found a similar AGS rate as the 3-day feeding regimen. Thus, a soy-based diet drastically influences seizure susceptibility in mice.

Purina-type lab chows are grain based with their protein content derived from soybeans (37). Soybeans are rich in phytoestrogens, “plant estrogens,” which can be transferred to offspring through the placenta as well as maternal milk. The protein source for the soy-free diet was casein instead of soybean-based, and thus, it did not contain any phytoestrogens. To determine if the AGS phenotype resurfaced in mice conceived on soy-free diet and later exposed to soy, Tg2576 mice born to parents maintained on the soy-free diet were weaned onto Purina 5015 at P18 and tested for seizures at P21. There was a statistically significant increase in seizures (36%, *P* ≤ 0.05) in the Tg2576 fed Purina 5015 for 3 days. We hypothesized that soy phytoestrogens were the seizure-promoting constituent in the Purina 5015. Soy protein is rich in a type of phytoestrogen called isoflavones, which are bioactive compounds structurally similar to the female hormone estrogen. Isoflavones can exert biological activity by mimicking the effects of mammalian estrogens and thus disrupt the endocrine cycle. The three most prevalent isoflavones in soy are genistein, daidzein, and glycitein. Hence, we tested the effects of individual isoflavones on seizures by supplementing the soy-free diet with the two most prevalent isoflavones, genistein, or daidzein, at 0.75 g/kg feed, which is within the concentration range of isoflavones naturally found in soy products (39, 40). Mice were bred on the soy-free diet and at P18 weaned onto daidzein- or genistein-supplemented diets for 3-days prior to AGS testing at P21. Daidzein significantly increased wild running in wild-type mice (39%, *P* ≤ 0.01), although 3 days feeding was not sufficient to induce a statistically significant increase in AGS (21%, *P* ≤ 0.1). Chronic dosing studies are currently underway. Genistein, or the combination of genistein and daidzein, did not alter wild running, AGS, or death rates suggesting that genistein may counteract the seizure-promoting effect of daidzein and that the ratio of the isoflavones in soy may determine seizure propensity (38).

In addition to our studies, the Hosseini laboratory in Iran has studied the effect of soy extract on pentylenetetrazol (PTZ)-induced seizures in rats (41, 42). First, they treated rats with repeated low doses of PTZ (40 mg/kg body weight) over 14 days in conjunction with low (20 mg/kg body weight) and high (60 mg/kg body weight) doses of soy extract 30 min before each PTZ injection. Both the low and high doses of soy extract elicited higher seizure scores in ovariectomized (OVX) rats as well as significantly decreased latency times to minimal clonic seizures (MCS) and generalized tonic-clonic seizures (GTCS) (42). Second, they treated male, female, and OVX female rats with high and low doses of soy extract for 14 days followed by injection of a single dose of PTZ (90 mg/kg body weight). They found that the soy extract decreased the latency times to MCS and GTCS in the male and OVX female rats but not in the female rats (41).

A literature search of the terms “soy” and “seizure” produces very few studies. In humans, there are several case reports involving infants with seizure phenotypes in response to consumption of a defective soy formula (Israel study) or who developed micronutrient deficiencies due to soy formula (Canadian study). The Israel study was a follow-up study of seven Israeli, female children, age 5–6 years, with severe epilepsy as a result of thiamine deficiency in infancy caused by a defective soy-based formula (43). Their findings indicated that severe infantile thiamine deficiency might result in epilepsy. The seizures were refractory to most antiepileptic drugs with four children having uncontrolled seizures and all children exhibiting mental retardation and brainstem dysfunction. The Canadian study reported on three case articles of infants presenting with hypocalcemic seizures during the winter months while being fed soy formula (44). The study population included three infants with different ethnic backgrounds and living in different geographical regions of Eastern Canada. All of the subjects were male and 6 week- to 2-months old and presented with hypocalcemic seizures attributable to vitamin D deficiency. Laboratory results were consistent with vitamin D deficiency despite receiving daily recommended intake levels.

There have been studies examining cognitive and reproductive development in infants fed soy-based infant formula. Malloy and Berendes tested 9- to 10-year-old-children who had been fed soy-based infant formula or human milk during their first year of life and found no difference in IQ, behavioral problems, learning impairment, or emotional problems (45). A study by Strom and colleagues surveyed adults age 20–34 years old who had participated as infants in controlled feeding studies between 1965 and 1978 (46). The study population included 811 subjects including males (*n* = 120) and females (*n* = 128) who had been fed soy-based infant formula. The outcomes were that no correlation was found between infant formula use and education level, but women in the soy cohort reported longer duration of menstrual bleeding (about 8 h) and greater discomfort with menstruation. The soy cohorts also had a higher reported use of asthma or allergy drugs and greater tendency toward sedentary activities. The consumption of soy-based infant formula has also been associated with breast development (47) and premature thelarche (48).

There have been several rodent studies assessing the effect of soy phytoestrogens on seizures, which are described above. The problem with studying soy in rodents is that they metabolize soy isoflavones differently from humans. Thus, the data may not be generalizable between species. It should be noted that infants can efficiently digest, absorb, and excrete genistein and daidzein from soy-based infant formulas (49). Urine was collected from disposable diapers (3–5 diapers worn during a 24-h period) of infants (4 received soy-based formula and 25 received cow milk-based formula). Isoflavones were extracted from the diapers every 1–2 weeks from a starting age of 2–6 weeks and continuing until 16 weeks of age and detected by HPLC. Isoflavone (genistein plus daidzein) levels remained constant at 3.2 ± 0.2 mg/kg body weight regardless of age. Rodents conjugate isoflavones less efficiently and thus have higher circulating concentrations of biologically active forms (50). With this caveat noted, studies in rats and mice have demonstrated that soy increases seizure propensity. In addition to the aforementioned rodent studies, the effects of soy have been studied in monkeys and *in vitro*. Dietary soy is associated with epigenetic changes in monkeys such that overall methylation in liver and muscle tissue was increased when switching from a soy-based to casein-based diet (51). At high doses, genistein and daidzein are toxic to primary neuronal cultures (52).

Overall, a soy-based diet significantly increases seizure propensity in genetically susceptible mice and daidzein is likely a contributing factor. The amount of daidzein consumed per body weight per day by juvenile mice is comparable to the daily isoflavone intake of infants fed soy-based formula (34, 53) suggesting that these findings could have important clinical relevance.

Hence, we conducted a retrospective analysis of seizure incidence in autistic children fed soy- versus casein-based infant formula. Seizures are a prevalent phenotype in autism (21–38%) (54, 55). We utilized medical record data from the Simons Foundation Autism Research Initiative – Simons Simplex collection (SFARI-SSC) to assess seizure incidence in autistic children fed soy-based versus other infant formula. There were data available for 1949 subjects (87% males). We found a 2.6-fold increase in the incidence of febrile seizures and a 4.8-fold increase in the incidence of simple partial seizures in autistic children fed soy formula (56) (Table 1). The soy-based formula was not associated with statistically higher rates of infantile spasms, atonic (drop attack), grand mal (generalized tonic clonic), petit mal (absence), or complex partial seizures. There was a 2.1-fold increased incidence of epilepsy. In aggregate, these data demonstrate that a soy-based diet is associated with increased seizure incidence in both mouse models of neurological disease and in autistic children. These data raise important questions regarding the neurological side effects of a soy-based diet during postnatal development.

**Table 1:** **Prevalence of seizures in autism subjects dependent on soy formula**.

Phenotype	Soy	Non-soy
Febrile seizures (%)	4.2**^††^	1.6
Infantile spasms (%)	0.60	0.063
Atonic seizures (%)	0.30	0.13
Grand mal seizures (%)	1.2	1.9
Petit mal seizures (%)	3.3	2.0
Simple partial seizures (%)	1.2^†^	0.25
Complex partial seizures (%)	0.60	0.38
Epilepsy diagnosis (%)	3.6*^†^	1.7

*Minimum number of subjects per cohort = 330 (soy) and 1563 (non-soy)*.

*P ≤ 0.05 and **P ≤ 0.01 as determined by the Pearson’s uncorrected chi-squared test.

*^†^P ≤ 0.05 and ^††^P ≤ 0.01 as determined by Fisher’s exact test (two-tail)*.

We also assessed developmental milestones and autism testing scores in the SFARI autism population dependent on soy-based infant formula. There were no statistically significant differences in developmental milestones (age first walked, age of first single word, or age of first phrase) dependent on soy or non-soy-based infant formula use. We did find exploratory associations between the consumption of soy-based infant formula and several autistic behaviors as assessed by sub-score and line-item analysis of the aberrant behavior checklist (ABC), autism diagnostic interview-revised (ADI-R), and autism diagnostic observation schedule (ADOS) (57). It is important to note that these findings were exploratory in nature as the SFARI data collection protocol was neither specifically designed to assess the effects of infant formula on autistic behaviors nor powered to detect multiple hypotheses based on line-item analyses of diagnostic tests.

There are limitations to the retrospective human data described above including declarative data regarding the epilepsy diagnoses, lack of data regarding potentially confounding issues, and less female subjects than male. A diagnosis of epilepsy was defined as either a specific report of epilepsy on the ADI-R or at least two seizures on the medical record history report, which are based on parental recall. A neurologist did not verify the diagnoses; however, NINDS considers detailed medical history reports of seizure history one of the best methods available to identify if a person has epilepsy as well as the type of seizures. Recall bias regarding infant formula usage is not an expected problem as parents typically switch formulas for very specific reasons such as gastrointestinal problems or allergies. There are confounding issues associated with the consumption of soy-based infant formula that will need to be addressed in future, prospective studies such as the reasons the infants were fed soy-based formula and the duration of feeding with the soy-based formula. It is possible that soy is a surrogate marker for an underlying condition that lowers seizure threshold. For example, infants that are fed soy-based infant formula due to cow milk protein allergies could be more vulnerable to illnesses associated with fever-induced convulsions. We can not make a definitive conclusion regarding this scenario based on retrospective data; however, in the SFARI study population utilized for our analysis, no subjects reported both allergies and febrile seizures. In accordance with current autism prevalence rates, there were significantly less female subjects than males in the study. Despite the lower number of females, the use of soy-based infant formula was associated with febrile seizures in females. A larger female cohort is required to confirm whether the use of soy-based infant formula is associated with epilepsy comorbidity in autistic girls.

## Implications of Soy-Based Infant Formulas on Childhood Neurological Development

The presented data suggest that the consumption of soy-based diets is associated with reduced seizure threshold in several mouse models of neurological disease as well as in a vulnerable population of children diagnosed with autism. These results require prospective evaluation regarding the effects of soy on childhood development particularly in infants genetically predisposed to seizure disorders. Many developmental disabilities are co-morbid with seizures and epilepsy including FXS, autism, and attention deficit/hyperactivity disorder (ADHD).

Fragile X syndrome is the most common form of inherited mental retardation and the leading known genetic cause of autism. This X chromosome-linked disorder is clinically characterized by highly variable intellectual disability (overall IQ < 70), autistic-like behavior, seizures, macrocephaly, and macroorchidism (58). FXS results from a mutation in a single gene on the X chromosome, *FMR1*. In the majority of cases, a >200 copy trinucleotide (CGG) repeat expansion in the 5′-UTR of the *FMR1* gene (59) is associated with transcriptional silencing of the *FMR1* promoter and loss of expression of fragile X mental retardation protein (FMRP) (60). FMRP is a multi-functional mRNA binding protein that is involved in the transport, localization, and translational regulation of mRNA ligands and is required for normal dendrite development. FMRP expression is absent or greatly reduced in FXS and many FXS phenotypes are manifested in *Fmr1^KO^* mice, which lack expression of FMRP. In the preceding section, we demonstrated an increased incidence of AGS in a mouse model of FXS in response to a soy-based diet. FXS is a family of disorders also including the *FMR1* premutation disorders fragile X-associated primary ovarian insufficiency (FXPOI) and fragile X-associated tremor/ataxia syndrome (FXTAS). There is an increased prevalence of seizures in boys with the *FMR1* premutation co-morbid with autism spectrum disorders (ASD) (61).

Autism is a cluster of complex neurobiological disorders that normally present in the second or third years of life. The core features include impairments in social interaction and communication and repetitive stereotyped behavior. Many autistic children are mentally retarded and half exhibit marked delay in motor milestones. ASD are estimated to occur in 1 in 88 children with prevalence 4.7-fold higher in males (62). The etiology of autism is not known but genetic as well as environmental factors likely affect the severity of symptoms (63–65). For example, autism is highly co-morbid with other developmental disorders such as FXS where 67% of males and 23% of females meet the diagnostic criteria for ASD (66). Epilepsy is highly co-morbid in autism with a prevalence of 21.4% in autistic subjects with intellectual disability and 8% in subjects without intellectual disability (67). EEG abnormalities were found in 31% of children with ASD (68), and are associated with language disorders (69). It has been proposed that epilepsy drives autism in neurodevelopmental disorders (70, 71).

Attention deficit/hyperactivity disorder is the most common neurobehavioral disorder diagnosed in children with a worldwide prevalence of 5.3% (72) and a national prevalence of 9.0% (73). ADHD is characterized by in attention, impulsivity, and hyperactivity. The diagnostic and statistical manual of mental disorders-fourth edition (DSM-IV) diagnosis requires the presence of six out of nine specific behavioral and functional symptoms of inattention or hyperactivity/impulsivity for a duration of at least 6 months, with onset before age 7 years. ADHD is highly co-morbid with ASD and epilepsy. Up to 70% of ASD cases (74) and 38% of children with epilepsy exhibit ADHD (75–77). ADHD is significantly more common among children with newly diagnosed epilepsy than among controls suggesting that there is a common antecedent for both conditions (78, 79).

Several factors could contribute to the comorbidity of epilepsy with these developmental disorders, such as underlying brain pathology, genetic susceptibility genes, and/or environmental and dietary factors that exacerbate epileptiform activity. It is estimated that there are over 350 autism susceptibility genes (80). Dietary factors remain less well characterized. The prevalence of epilepsy in patients with celiac disease is 5.5% (81). Elevated levels of manganese are associated with neurocognitive deficits (82, 83), and it has been proposed that soy-based infant formula may cause ADHD due elevated manganese content (84). We hypothesize that soy phytoestrogens are a dietary factor that increases epileptiform activity, which leads to the development of ADHD and autism. In Israel where there is widespread use of soy-based formula without clinical indications (70% of children receive soy for >6 months) (85), there is a high rate of ADHD (12.6%) (86). In the SFARI autism population described in the previous section, we observe a statistically significant increase in ADHD with the consumption of soy-based infant formula (6.7% soy, 3.9% non-soy, 1.7-fold increase, *P* = 0.04). Thus, the consumption of soy-based infant formulas may be altering neuronal excitability and contributing to the increased incidence and/or severity of neurological disorders.

## FXS and mGluR Signaling

Seminal work by Drs. Kim Huber and Mark Bear has shown that excessive signaling through mGluR_5_ contributes to many of the psychiatric and neurological aspects of FXS (87). Genetic reduction of mGluR_5_ in an *Fmr1^KO^* background (88) or pharmacological treatment with an mGluR_5_ inhibitor (89–91) rescues many FXS and autistic phenotypes. mGluRs belong to the G-protein-coupled receptor superfamily. There are eight identified subtypes of mGluR that have been classified into groups based on sequence homology and signaling properties. Group 1 (mGluR_1_ and mGluR_5_) are generally postsynaptic in location, couple to G_q_, and activate phospholipase C. mGluR_5_, which signals through FMRP, has been the major target of drug discovery for FXS over the past decade (87). The “mGluR theory of FXS” proposes that FMRP binds to synaptic mRNAs and represses their translation. Upon mGluR_5_ activation, FMRP is dislodged or inactivated, and translation proceeds. This accounts for “regulated translation” at the synapse. In the absence of FMRP, as in FXS, mGluR_5_-mediated translation is constitutive and unregulated. Over the past two decades, there have been pivotal advances regarding the identification of downstream signaling molecules and translational targets in the mGluR_5_/FMRP pathway by academia as well as the development of numerous robust mGluR_5_ inhibitors by pharmaceutical companies. However, there is a paucity of knowledge regarding the upstream activators and environmental factors that stimulate mGluR_5_ signaling. Based on our preliminary data presented above, we hypothesize that estrogenic compounds in soy stimulate mGluR_5_ signaling and thus contribute to FXS pathogenesis.

## ER Signaling at the Synapse

The major “female” and “male” steroid hormones are classified as estrogens and androgens, respectively. Both classes of hormones are found in both genders but in different quantities. The most predominant and potent estrogen is estradiol. Estradiol can be synthesized by the endocrine glands and secreted into the bloodstream and thereby enter and stimulate target tissues including the brain. Estradiol can also be synthesized locally within the brain (92) *de novo* from cholesterol or derived from testosterone by P-450 aromatase, also known as estradiol synthase, through an aromatization reaction. Hippocampal neurons produce P-450 aromatase and generate estradiol (93). Estradiol acts on both the alpha and beta forms of ER, ERα and ERβ, which are primarily localized in the cell nucleus where they act as ligand-regulated transcription factors by binding to specific estrogen response elements (ERE) on DNA, i.e., “classical ER signaling.” However, accumulating evidence demonstrates that ERα and ERβ are also found at cell membranes where they interact with mGluRs to initiate cell signaling, i.e., “non-classical ER signaling.” The Mermelstein laboratory has proposed the “ER/mGluR hypothesis” to explain the rapid membrane actions of estrogen in the nervous system. Their theory purports that direct protein–protein interactions between ER and mGluRs allow estradiol to signal through mGluRs. Upon estradiol binding to the ER, the ER alters the conformation of mGluR, resulting in activation of the downstream G-proteins and second messenger signaling without the requirement for glutamate (94). Both ERα and ERβ are detected on dendritic spines and axon terminals; however, ERβ has more widespread localization to extranuclear sites suggesting that this isoform may be more important in mediating rapid estrogen signaling at membranes (95).

Soy phytoestrogens can act as endocrine disrupting chemicals (EDC) (96), and the developing fetus and neonate are particularly vulnerable to their effects. EDC bind to ER and induce ER-mediated gene expression and altered cell signaling. Peri- and postnatal exposure to EDC are expected to disrupt the hypothalamic–pituitary–gonadal axis (HPG axis), which is critical for the development of the reproductive and immune systems. It has been hypothesized that premature activation of the HPG axis is the cause of growth impairment in FXS (97). Thus, dietary exposure to high levels of soy phytoestrogens during infancy could negatively impact growth and development.

There is a paucity of studies regarding the effects of phytoestrogens on neuronal excitability. It is known that genistein is a broad-spectrum tyrosine kinase inhibitor, and tyrosine kinases modulate synaptic plasticity and ion channel function. Genistein has been shown to decrease neuron excitability in *Aplysia* sensory neurons (98), 3,5-DHPG-induced membrane potential oscillations in striatal cholinergic interneurons (99), and excitability of capsaicin-sensitive neurons from cultured rat trigeminal ganglion neurons (100). Daidzein, which is a structural analog of genistein, that does not possess protein tyrosine kinase inhibitor activity, also decreases excitability but to a lesser extent than genistein (100). Much remains to be learned regarding the effects of daidzein and other phytoestrogens on neuronal excitability.

## Melding the Bear “mGluR Theory of FXS” with the Mermelstein “ER/mGluR Hypothesis”

**Figure 2:**
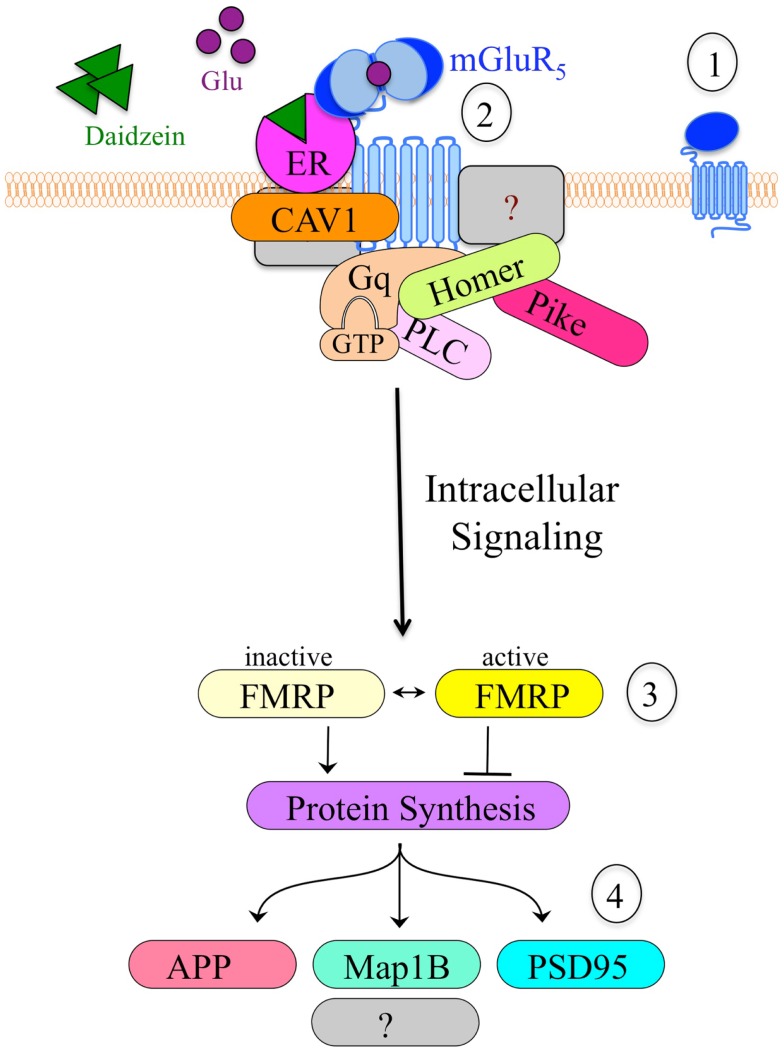
**Model of the hypothetical estrogen-induced signaling pathway in neurons**. (1) mGluR_5_ is a primary target for drug development in FXS. (2) Cell stimulation causes clustering of mGluR_5_ receptors and altered interactions with scaffolding proteins such as ER, caveolin and Homer, which likely alter downstream signaling events. Daidzein or other estrogenic compounds act as ER agonists. (3) mGluR_5_ is known to signal through FMRP, a translational repressor. The absence of FMRP in *Fmr1^KO^* cells results in constitutive, unregulated protein synthesis in response to mGluR_5_ stimulation. (4) Altered mGluR_5_/FMRP signaling modulates the synthesis of numerous synaptic proteins including APP, microtubule-associated protein 1B (Map1B), and postsynaptic density protein 95 (PSD95). Excessive production and accumulation of these synaptic proteins contributes to elevated epileptiform activity and FXS pathology.

An important, unanswered question regards how soy and/or soy isoflavones affect neuron function. Both genistein and daidzein inhibit GABA_A_R (101), which is the major inhibitory receptor in the brain with roles in seizures, hyperactivity, learning and memory, and sleep/wake cycles. Alternatively, or perhaps concurrently, soy isoflavones could affect neuronal function through mGluR_5_ signaling. We are exploring the hypothesis that soy phytoestrogens such as daidzein promote altered mGluR_5_/ER/scaffolding protein interactions, resulting in activated mGluR_5_ signaling, which leads to increased epileptiform activity. This hypothesis developed from our data described above demonstrating that dietary soy consumption is associated with increased seizure incidence in *Fmr1^KO^* mice (38) and in autistic children (56). These data in conjunction with published reports demonstrating the interaction between ER and mGluRs (102–105) suggest that soy phytoestrogens could exacerbate mGluR_5_ signaling through an ER-dependent mechanism. Excessive levels of estrogenic compounds are predicted to increase mGluR_5_ activation and downstream signaling, particularly in FXS where the FMRP translational brake is absent. Thus, we propose that coupling the Bear “mGluR theory of FXS” with the Mermelstein “ER/mGluR hypothesis” provides a plausible mechanism through which estrogenic compounds such as soy phytoestrogens lower seizure threshold (Figure 2).

## Alternative Hypotheses

There are alternative hypotheses, which could explain how soy lowers seizure threshold including non-neuronal targets. For example, understanding how the bacteria in the gut can affect the brain and disease development is an emerging area of important research. Bacteria in the gut have the potential to communicate with the brain through the vagus nerve, by modulating the immune system and/or by synthesizing novel neurotransmitters. It is known that soy isoflavones affect the development of the intestines as well as the make-up of the intestinal microbiota (106). There have been two studies investigating the influence of soy-based infant formula on the gut microflora in infants and/or children. The first study found increased equol excretion in the soy group (19%) compared to controls (5%) with elevated *Bifidobacteria, Bacteroides*, and *Clostridia* bacteria in fecal samples from the soy group (107). The second study found elevated *Bifidobacteria* species (*B. adolescentis* and *B. infantis*), which were not detected before commencing the soy-based formula (108). *Bifidobaterium, Bacteriodes*, and *Clostridium* are among the human intestinal bacteria that can produce *S*-equol (109, 110), which is the biologically active metabolite of daidzein. Intestinal bacteria transform daidzein to equol in humans that are equol producers. In Japan, Korea, and China, up to 80% of people are equol producers, but as few as 25% of people in North America and Europe can biotransform daidzein into equol (110). Equol modulates expression of the BRCA1 and BRCA2 breast cancer genes through an epigenetic mechanism, resulting in decreased methylation (111). The effect of equol on the methylation of neuronal genes has not been studied.

DNA and protein constituents of soy may also affect brain function. It was long thought that large macromolecules do not pass directly from the digestive tract to the circulatory system. Emerging evidence is proving this paradigm false. Meal-derived DNA fragments that are large enough to carry intact genes can avoid degradation by the stomach acid, enter the human circulatory system, and be detected in blood plasma (112). In addition to plant DNA, >90% of soybeans are genetically modified (GM) and carry bacterial genes that can code for toxic proteins. It has been estimated that if there were a 1 in a billion event of bacterial transformation of GM DNA into gut bacterium and that with 1E15 bacteria in the human gut, one would potentially have 1 million transformed bacteria in the intestines (113). These transformed bacteria could constitutively express toxic proteins that could damage the intestinal lining and cause disorders like celiac disease in which peptides enter the circulatory system, stimulate antibody production, and lead to autoimmune reactions that can affect neurological function. The second most common GM trait codes for a built-in pesticide produced by the soil bacterium *Bacillus thuringiensis* (Bt).

Besides the macromolecular content of food (DNA and protein), chemical contaminants associated with soy have strong potential to affect neurological and gut function. There is a strong correlation between glyphosate usage and modern disease incidence (114). Moms Across America and Sustainable Pulse conducted a small study of lactating mothers across the United States. They found high levels of glyphosate (76–166 mg/L) in 3 out of 10 breast milk samples. Glyphosate is sprayed on GM crops where it acts as both a pesticide and herbicide, as well as on other crops where it serves as a drying agent. This chemical that accumulates in the soil is not easily degraded and is a known antibiotic and endocrine disruptor. The Moms Across America was a small, grassroots study to encourage future, controlled, scientific investigation in this area. They could not afford to do a sensitive HPLC analysis; thus, the prevalence of lactating mothers with glyphosate in their breast milk could be higher than reported. There is one anecdotal report of the negative detection of glyphosate coinciding with the disappearance of inflammation and autism symptoms in an 8-year-old boy after 6 weeks on an organic diet and 2 weeks of reverse osmosis filtered water that tested negative for glyphosate.

Finally, substantial evidence suggests that activation of the immune system is associated with epilepsy (115, 116), and consumption of soy and soy phytoestrogens is associated with activation of the immune system. Specifically, soy-based diets alter cytokine production through an ERα-dependent pathway (117). Mice fed high doses of daidzein (20 and 40 mg/kg/day) for 7 consecutive days exhibit enhanced non-specific immunity, humoral immunity, and cell-mediated immunity (118). In addition, exposure to environmental estrogenic compounds is implicated in the increased prevalence of autoimmune disorders (119). Thus, soy phytoestrogens and/or estrogenic pesticides associated with soy may indirectly affect seizure propensity through modulation of the immune system. This alternative hypothesis may occur through disruption of the blood–brain barrier (BBB). Recent work from Bargerstock and colleagues has shown that peripheral immunity may regulate changes in the BBB through a BBB integrity biomarker, S100B, which is detected in blood when the BBB is compromised. Pilocarpine-induced status epilepticus is also associated with increased circulating levels of S100B, which is thought to trigger an autoimmune reaction (120). The original finding that prompted the development of this hypothesis paper was an association between febrile seizure incidence and the consumption of soy-based infant formula (56). Fevers increase brain temperature in part through the release of inflammatory cytokines, which are likely involved in the generation of febrile seizures (121). Thus, interactions between the immune and nervous systems via a compromised BBB may affect seizure propensity with dietary factors such as soy acting as an immune system trigger.

## Concluding Remarks

In summary, soy infant formula has been used in the United States since 1909 as an alternative for infants allergic to cow’s milk. Published estimates of formula intolerance range from 2 to 7.5%; yet, about a quarter of infants are fed soy-based formulas suggesting that non-standard, soy-based formulas are used excessively (122). Understanding the health consequences of soy phytoestrogens and modulating intake of these compounds during pregnancy and infancy could potentially decrease the development and/or exacerbation of childhood neurological disorders. There have been no clinical trials examining the effects of soy-based formulas on seizures or neurological development in infants with developmental disabilities. These vulnerable groups may be more susceptible to seizure-promoting ingredients in the diet. Prospective studies are required to validate the rodent and human analyses described herein. There are numerous potentially confounding factors that need to be addressed, most notably, the lack of data regarding the age of initiation and the duration of feeding with soy-based infant formula. Studies examining the health effects of soy and soy phytoestrogens are further complicated by the fact that Monsanto marketed GM soy in 1995. Glyphosate-tolerant soybeans were genetically engineered to express the 5-enolpyruvyl shikimic acid-3-phosphate (EPSP) synthase gene from *Agrobacterium* sp. (strain CP4), which infers resistance to Roundup^®^ herbicide. Currently, 93% of the soybean crops in the United States are GM. Glyphosate, the active ingredient in Roundup^®^, is a major environmental toxin implicated in the increased incidence of autism, Alzheimer’s disease, cancer, and many other diseases (114). Thus, considering the lack of existing data regarding the long-term neurological consequences of consuming a soy-based diet during infancy, particularly in children with developmental disabilities, alternative formulas should be considered when clinically indicated.

## Human Subjects

All studies conducted by the author in rodents or by retrospective analyses of human medical record data were performed in accordance with institutional and national guidelines and regulations. The mouse studies were conducted under an approved University of Wisconsin-Madison animal care protocol administered through their Animal Resource Center. The institutional review protocol governing the Simons Simplex collection was approved by the Institutional Review Board at Columbia University Medical Center. Written informed consent was provided by all guardians or research subjects. The privacy of participants was protected by using global unique identifiers. The research protocol for using the Simons Simplex collection in the studies described herein was approved by the Human Research Protection Program at the University of Wisconsin-Madison, which determined that the study qualified for exemption.

## Conflict of Interest Statement

The author declares that the research was conducted in the absence of any commercial or financial relationships that could be construed as a potential conflict of interest.
